# Urban bat pups take after their mothers and are bolder and faster learners than rural pups

**DOI:** 10.1186/s12915-021-01131-z

**Published:** 2021-09-07

**Authors:** Lee Harten, Nesim Gonceer, Michal Handel, Orit Dash, H. Bobby Fokidis, Yossi Yovel

**Affiliations:** 1 School of Zoology, Faculty of Life Sciences, Tel Aviv University, 69978 Tel Aviv, Israel; 2 Department of Biology, Rollins College, P.O. Box 874601, Winter Park, Florida 32708 USA; 3 Sagol School of Neuroscience, Tel Aviv University, 69978 Tel Aviv, Israel; 4 Wissenschaftskolleg zu Berlin, Berlin, Germany

**Keywords:** Personality, Urbanization, Maternal effects, Risk-taking

## Abstract

**Background:**

Urbanization is rapidly changing our planet and animals that live in urban environments must quickly adjust their behavior. One of the most prevalent behavioral characteristics of urban dwelling animals is an increased level of risk-taking. Here, we aimed to reveal how urban fruitbats become risk-takers, and how they differ behaviorally from rural bats, studying both genetic and non-genetic factors that might play a role in the process. We assessed the personality of newborn pups from both rural and urban colonies before they acquired experience outdoors, examining risk-taking, exploration, and learning rates.

**Results:**

Urban pups exhibited significantly higher risk-taking levels, they were faster learners, but less exploratory than their rural counterparts. A cross-fostering experiment revealed that pups were more similar to their adoptive mothers, thus suggesting a non-genetic mechanism and pointing towards a maternal effect. We moreover found that lactating urban mothers have higher cortisol levels in their milk, which could potentially explain the transmission of some personality traits from mother to pup.

**Conclusions:**

Young bats seem to acquire environment suitable traits via post-birth non-genetic maternal effects. We offer a potential mechanism for how urban pups can acquire urban-suitable behavioral traits through hormonal transfer from their mothers.

**Supplementary Information:**

The online version contains supplementary material available at 10.1186/s12915-021-01131-z.

## Background

The existence of consistent intra-species, inter-individual differences in behavior, often referred to as personality traits [1, 2], is now well established in a wide range of animal taxa [1–12]. The notion that this behavioral variation is merely a result of statistical “noise”, is progressively changing to the understanding that such variability may be in itself adaptive and thus maintained by natural selection [2, 6, 13–20]. One interesting idea is that intra-species behavioral variation is adaptive because of the heterogeneity and dynamics of available living habitats [21–23]. Indeed, evidence suggests that behavioral traits in various groups such as insects, reptiles, birds, and mammals diverge along environmental gradients [21, 24–30]. Behavioral gradients can be a result of exposure to local stimuli, learning, environment-specific selection pressures, or individual differences in perception (e.g., threats). Increasing evidence indicates that an animal’s personality shapes how it exploits novel environments. The rapid increase in urbanization [31] is an especially good example where behavioral variability can be advantageous. Exploiting an urban environment often requires a specific set of skills, due to the distinctly different challenges, risks, and rewards presented by urban environments in comparison to the natural habitats where animal behavior has evolved [26, 28]. Indeed, one of the phenomena found repeatedly is that bolder individuals with a greater propensity to take risks colonize urbanized habitats more rapidly than shyer, less bold, and often also less aggressive individuals [2, 21, 32–36].

A major open question is how such urban-related behavioral traits are acquired. It remains unknown if they are a result of selection, of non-genetic inheritance, of self-experience and learning, or of a combination of the above, as different studies suggest [21, 24, 37–39]. Some studies examining the genetic basis of personality found that up to 30% of individual differences in behavior are genetically inherited [14, 29, 40]. Dingemanse et al. (2002) for example reported that 30% of the variation in explorative behavior of great tits was attributed to their wild-caught parents [41]. Similarly, Alpine swifts resembled their genetic, but not foster parents in their anti-predator behavior [42]. Alternatively, a divergence of personality traits across environmental gradients might suggest non-genetic processes such as parental programming or individual experience-based learning [7, 29, 43, 44]. Maternal effects have been shown to influence the development of offspring behavioral traits in various ways [44–47]. Maternal effects include nutrient provisioning, hormone transfer, social interactions, or serving as a model to learn from [44, 48, 49]. For example, individual differences in maternal care and in stress reactivity are behaviorally transmitted between generations in rats [45], and the exploratory tendencies of young zebra finches are better predicted by the exploratory behavior of the foster than the genetic parents [29].

The Egyptian fruitbat, *Rousettus aegyptiacus*, successfully exploits human-altered landscapes and can be found abundantly both in urban and in rural environments in Israel. In a preliminary study, we revealed significant differences in the risk-taking behavior of fruitbats from rural and urban colonies with urban bats being bolder risk-takers (Additional file 1: Figure S1). Furthermore, these bats exhibit relatively long offspring dependency (up to 4 months), increasing the potential for different types of maternal influences on pup behavior. Taken together, this species presents a unique opportunity to detangle the determinants of personality traits across environmental gradients. We used common garden experiments, which remove the confounding effects of the rearing environment, in combination with cross-fostering experiments, to explore divergence in behavioral traits between urban and rural newborn pups and to explore whether these differences are innate or acquired.

## Results

### Urban and rural pups consistently differ in behavioral traits

In total, we carried out behavioral assays on 86 bat pups with no self-experience outdoors, across three years including 61 pups from four urban colonies and 25 from three rural colonies (Methods, Additional file 1: Table S1). We used the foraging box assay to assess bat personality (Methods). This assay has been successfully utilized by us in a preliminary study, showing behavioral differences between urban and rural adult fruitbats (Additional file 1: Figure S1). The setup consists of six identical plastic boxes placed on the floor, each with ample available fruit inside. A single bat at a time was allowed to explore the room for an entire night and it had to enter the boxes in order to eat.

Pups originating from urban and rural colonies differed consistently and significantly in risk-taking, exploratory, and learning behaviors:

1. *Risk-taking* was defined as the proportion of times an individual entered boxes after landing on them. Fruitbats typically show high vigilance when landing and hesitate before entering a box positioned on the ground as we have shown in the previous [50]. This hesitance is also typical for these bats when landing on low tree branches in the field [50].
2. *Exploration* was defined as the proportion of unique boxes entered by an individual throughout the experiment (the unique number of visited boxes divided by the total number of boxes). As all boxes contained ample food, in theory, a bat could have continued exploiting the first box it entered all night.

Note that risk-taking and exploration are not necessarily linked. An individual could land on six different boxes and enter them all, and thus be ranked an exploratory risk taker; it could land on a single box ten times and enter it once, thus ranked non-exploratory and hesitant, and it could show any combination of the two traits.

3) *Learning*: To test learning, we slightly changed the setup, now allowing access to the food only in one of the boxes, so that a bat that already experienced the previous setup had to learn that only one box offers food. We then measured the average error rate, i.e., the number of times a bat sampled wrong boxes, after discovering they are no longer rewarding, divided by its general activity (i.e., the sum of all landings).

**Fig. 1:**
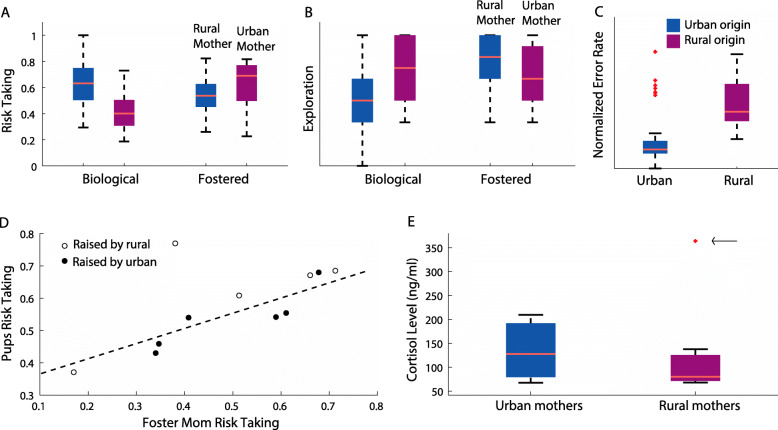
Inexperienced urban and rural pups consistently differ in their behavior. Boxplots show **A** risk-taking, **B** exploration, and **C** learning traits of the pups as a function of their origin: urban (blue) vs. rural (red) and their rearing (biological or fostered). **D** Cross-fostered pups resemble adoptive mothers in their risk-taking behavior. **E** Urban mothers’ milk contains higher levels of cortisol. Notice arrow pointing at the removed outlier rural mother. Box plot lower and upper box boundaries show the 25th and 75th percentiles, respectively, with the median inside. The lower and upper error lines depict the 10th and 90th percentiles, respectively. Outliers of the data are shown with red crosses

Urban-born pups exhibited greater risk-taking and faster learning than rural pups (Fig. 1A, C; *Risk-taking*: 0.62 ± 0.16 vs. 0.41 ± 0.14 box entries per landings, respectively, *R*^2^=0.58 *P* < 0.0001, mixed model GLM with risk-taking set as the response variable, the origin and fostering condition (i.e., biological, or foster mother) as fixed factors; and the bat’s ID and the year of the experiment as random effects, *n* = 86 bats). Results remained highly significant when excluding the cross-fostered bats (*n* = 11), which were only tested in year 3 (*P* < 0.00001; *learning*: 0.19±17 vs. 0.45 ±0.19 error rates, respectively, *R*^2^=0.72, *p* < 0.00001, mixed GLM with learning set as the response variable, the origin as a fixed factor and the bat’s ID as a random effect, *n* = 65). Both traits (risk-taking and exploration) were repeatable across trials (*R* values for Pearson’s correlation varied between 0.55 and 0.66 for risk-taking, Additional file 1: Figure S2A, *p* < 0.02 and between 0.6 and 0.86 for exploration, *n* = 48 individuals, Additional file 1: Figure S2B, *p* < 0.004). Urban pups were also less exploratory than rural pups (Fig. 1B; proportion of visited boxes − 0.73±0.22 vs. 0.58 ±0.23, for rural and urban pups respectively, *R*^2^=0.59, *p*=0.001, mixed model GLM as above, but with exploration set as the response variable).

The differences in both risk-taking and exploration were observed two years in a row, even when examining the data of each year separately (*P*< 0.0001 and *P*=0.0003 for risk-taking and *P*=0.009 and *P*=0.03 for exploration, mixed effect GLM as above, without the year random effect). As pups never experienced their environment of origin *independently*, we can rule out that these differences are a result of environment experiential effects.

Some of the traits were correlated with each other. Risk-taking was negatively significantly correlated with the error rates in the learning task and nearly significantly correlated with exploration, that is, pups more prone to take risks exhibited faster learning and less exploration (*R*= − 0.47, *P*=0.0001; *R* = − 0.19, *P*=0.07; respectively, Pearson’s correlation test). The negative correlation between the error rates in the learning task and exploration also approached significance (Pearson’s correlation test; *R* = − 0.22, *P*=0.08; respectively). Neither sex nor age at first experimental exposure, influenced the pups’ risk-taking, exploratory behavior, or learning (sex: *P*=0.37, age: *P*=0.12; sex: *P*=0.07, age: *P*=0.8; sex: *P*=0.9, age: *P*=0.23; respectively; mixed model GLM, as above but with sex\age added as fixed factor.)

### Pups are more similar to their adoptive than to their biological mothers

To examine whether the behavioral differences between urban and rural pups are innate or acquired post-birth through maternal effects, we carried out cross-fostering experiments, where we attached pups from urban colonies to mothers from rural colonies who reared them and vice-versa (*n*=11 mother-pup pairs). The cross-fostering was performed during the first 3 weeks of the pups’ lives in which they are constantly attached to their mothers’ nipple. Pups remained with either their biological or adoptive mothers’ care for another ca. 80 days prior to participating in the behavioral assays, thus providing the opportunity for maternal effects to take place.

Cross-fostering significantly affected pups’ risk-taking behavior, suggesting a maternal role in the acquisition of this behavioral trait. Rural pups raised by urban mothers were more prone to take risks than rural pups raised by rural mothers (Fig. 1A, cross-fostering had a significant effect on risk-taking, *P*=0.003 for the effect of the fostering condition, mixed effect GLM with risk-taking set as the response variable, the origin and rearing conditions as fixed factors, and the bat’s ID and year of experiment set as random factors, *n*= 86 bats, 156 trials). Post hoc contrasts showed that fostered rural bats exhibited significantly higher risk-taking than biologically raised rural pups, whereas the difference between fostered and biologically raised urban pups only approached significance (*P*< 0.0001 and *P*=0.056; respectively, the critical *p* value following a Bonferroni correction was 0.025). Furthermore, we found that cross-fostered pups resembled their adoptive mother and not their biological mothers in their levels of risk-taking. Pups’ risk-taking was significantly positively correlated with that of their adoptive mothers, (Fig. 1D *R*=0.65, *p*=0.02, Pearson’s correlation; *n*=11 pairs). Pups’ risk-taking was negatively (non-significantly) correlated with that of their biological mothers (*R* = − 0.59. *p* = 0.07, Pearson’s correlation; *n*=7 pairs).

Although fostering showed the same patterns for exploration (Fig. 1B), the effect was only significant for the interaction between the origin of the pups and the fostering condition suggesting that pups from rural colonies changed significantly more under cross-fostering. (*P*=0.07 for the fostering condition and *p*=0.04 for the interaction between the origin and the rearing condition, mixed model GLM as above but with exploration as the response variable). In the case of the exploration, there was no correlation between the pups and either the biological or the foster mothers (*R*=0.22, *P*=0.5; *R*=0.05, *P*=0.9; Pearson correlation test, respectively).

Finally, to examine the possibility of a hormonal maternal influence on pup behavior, we examined cortisol levels in the milk of 31 lactating mothers (17 urban and 14 rural, see the “Methods” section). Urban bats had higher levels; significantly higher when removing one extremely rural outlier that was more than 2 standard deviations from the mean (132±56 vs. 92±26 cortisol (ng/ml), for urban and rural mothers respectively; Fig. 1E, *P*=0.4 without removal and *P*< 0.02 with the removal of one point, *T* test).

## Discussion

We set out to examine the determinants of the personality differences between adult Egyptian fruitbats from urban and rural colonies (Figure S1). We tested newborn pups in a common garden experiment, which eliminates confounding effects of self-experience and in a cross-fostering experiment aiming to disentangle the contribution of genetic and maternal effects on offspring personality traits.

Bat pups originating from urban and rural environments significantly and consistently differed in their risk-taking, exploratory, and learning even though they were caught prior to developing flight abilities, and thus had no opportunity to independently experience the environment. In line with previous studies on a wide range of species (e.g. *1*–*6*) urban bat pups exhibited significantly higher risk-taking tendencies than their rural counterparts.

Previous findings indicate that the roosting environment (urban or rural) of Egyptian fruitbats does not necessarily determine their foraging grounds of its inhabitants. However, although rural-dwelling Egyptian fruitbats spent on average 45% of their time foraging in settlements very few cases of urban-dwelling bats were observed consistently foraging in the countryside. Thus, the foraging and roosting ecology of urban and rural dwelling populations remain substantially different. Moreover, bats that roost in urban environments are more exposed to urban challenges than bats that roost in rural environments spend a few hours foraging in cities [51]. Indeed behavioral differences between urban and rural populations have been established both in the wild and captivity for this species (Additional file 1: Figure S1, [51]). The propensity of risk-taking is probably greater in urban dwellers because of their need to better cope with the novel challenges associated with the urban environment [2, 21, 32, 33]. Urban pups were also significantly faster learners than rural pups, supporting previous evidence that bolder (i.e., risk-taking) individuals learn faster (e.g. voles*-12*, *13*). The ability to learn fast is thought to be especially advantageous in urban environments where environmental changes often outpace an animal’s ability to adapt [52]. Urban risk-taking pups were significantly less exploratory than rural pups. This result is seemingly not in line with previous studies where proactive bold, risk-takers are typically also more exploratory [5, 10]. However, exploratory tendencies are mostly measured in terms of speed of exploration, while here we measured whether individuals keep exploring after discovering a profitable resource. Our results thus might be similar to the findings that bold individuals also tend to be more rigid and routine-bound than shy counterparts, which might have led, in our case, to visiting the same boxes again and again and thus to being less exploratory according to our measurement [53].

After establishing that personality differences between urban and rural pups were not a result of environmental experience, we next used a cross-fostering paradigm to explore whether these differences were innate or acquired. We found that cross-fostering significantly influenced pups’ risk-taking and exploration, suggesting a maternal role in the acquisition of these behavioral traits. Rural pups raised by urban mothers exhibited higher risk-taking and lower exploratory tendencies than rural pups raised by their biological mothers and urban pups raised by rural mothers exhibited reduced risk-taking levels and more exploratory behavior. Furthermore, cross-fostered pups resembled their adoptive mothers and not their biological mothers in their risk-taking tendencies, further strengthening the maternal role in the acquisition of this behavioral trait. We thus suggest that the difference in risk-taking and to a lesser extent exploration between urban and rural populations is due to post-birth non-genetic maternal effects. Moreover, the significant correlation between the risk-taking levels of the adoptive mothers and the adopted pups suggests that risk-taking was influenced by some maternal effect and not by the cross-fostering manipulation itself or by social learning from conspecifics. Although we cannot completely rule out the potential role of conspecifics, the chances to learn from a pup that was non-volant for a substantial part of the period is low. This correlation also points against in-utero effects. Fostering mothers could influence pups through various mechanisms including social learning, hormone transfer, and other epi-genetic mechanisms. We find the social learning hypothesis less likely as pups did not have many opportunities to learn. In the relatively small colonies where the pups were held, mothers mostly perch near other bats or go down to the bowl of fruit to collect fruit. Of course, we cannot exclude the possibility that risk-taking mothers exhibit different behavioral patterns when performing these behaviors and that pups absorb and learn them. We find the hormonal hypothesis more likely and indeed our cortisol analysis suggests higher levels in urban bats, although more data is necessary to relate these levels with behavior. Hormonal levels, controlled via breast-feeding or elevated through maternal behavior have been shown to exert effects on offspring behavior in various species [44–46, 54–56].

Maternal cortisol-transfer can potentially have a profound impact on development; unfortunately, however, this area is understudied. The extremely high circulating cortisol levels reported for bat species [57–59] suggest that they would easily permeate all tissues. Thus, there is a good chance that even occasional urban-associated stressors may be sufficient to induce long-lasting changes in behavior. Furthermore, evidence from cows indicates that increased cortisol exposure is associated with increased permeability through the blood-milk barrier [60]; thus, there could be a positive feedback with cortisol dosing for the nursing young. The differences in cortisol levels we observe between the two populations might stem from various reasons unrelated to the ontogeny of pup behavior. For example, they might be related to metabolism. Cortisol is an energy mobilizing hormone firstly, and differences in metabolism could lead to such cortisol differences. Egyptian fruitbats have been shown to consume a greater variety of food sources per hour in urban environments. This may require increased cortisol levels in urban populations which in turn, might affect pup behavior. Interestingly, for both risk-taking and exploration, rural pups were more influenced than urban pups by the fostering manipulation. This suggests that if cortisol is the transmission mechanism, its presence during development affects behavior, but its relative absence has less effect. This is consistent with previous studies on how excessive concentrations of steroids cause changes in behavior. Our results are in line with several previous findings showing maternal effects on offspring personality [44]. For example, exploratory tendencies of young zebra finches are better predicted by the exploratory behavior of their foster parent rather than by their genetic parents [29]. Clearly, we cannot exclude some contribution of genetic predispositions to the behavioral traits that we studied. Indeed, conflicting results in the literature regarding the innate or acquired nature of behavioral traits (e.g., *17* vs. *47*), indicate that inheriting and acquiring individual-specific behavioral traits are not mutually exclusive. The mechanisms shaping pups’ behavior might depend on system-specific selection pressures promoting flexibility and variation of different traits within populations. An interesting idea in this respect is the distinction between selection pressures exerted on flying vs. terrestrial animals; where terrestrial animals are expected to be subjected to stronger selection pressures given their reduced capability to vacate urban habitats resulting in reduced gene flow [27, 61–63].

## Conclusions

In our rapidly changing world, it is crucial to understand what determines the success of certain individuals in urban environments. Specifically, are offspring of urban dwellers born adapted to urban environments? Our results join a substantial body of work exploring the influence of environmental [21, 24–30], genetic [14, 29, 40], and maternal effects [7, 29, 43, 44], on behavioral traits of offspring. But, rarely has the effect of urbanization on behavioral traits been examined while controlling for both the rearing environment [27] and maternal effects, and to our knowledge, never has cross-fostering been attempted in bats before. We highlight the importance of maternal effects as a mechanism generating and maintaining behavioral variation in heterogeneous habitats. We offer a potential mechanism explaining how urban pups can acquire urban-suitable behavioral traits through hormonal transfer from their mothers.

## Methods

### Study site, study species, and colonies

#### Permits

All experiments were approved by the TAU IACUC – permit number: 04-18-030. Bat capture was approved by the Israel National Park Authority.

#### Captive colonies and experimental rooms

Between September 2017–Jan 2018, April–May 2018, and April–May 2019, 86 Egyptian fruitbat pups were captured together with their mothers in natural rural and urban colonies and brought to the Zoological Garden at Tel Aviv University. Rural colonies consisted of natural caves outside settlements while urban colonies consisted of either caves or abandoned old buildings in the middle of the city (Additional file 1: Table S1). Rural colonies are positioned at least 10 km away from any city. Notably, roosting inside a city means much more interaction with humans and urbanization. To ensure that pups did not have any previous own navigational or foraging experience in their environment of origin we caught pups before reaching their volant stage, i.e., before reaching an age of 65 days or a forearm of ~ 74 mm [64]. We further validated for each of the captured pups that it cannot fly independently before releasing in its respective colonies. All ages were approximated based on their forearm, using an equation fitted to empirical data of 38, pups born in the lab between 2012 and 2019 [64].

##### Year 1—2017

Thirty five mother-pup pairs were captured at a single urban colony and brought to the lab prior to developing independent flight (average pup age was 51±27 days, 23 females, 12 males, Additional file 1: Table S1).

##### Year 2—2018

Thirty mother-pup pairs were captured at 2 rural and 4 urban colonies (Additional file 1: Table S1, *n*=15 urban; average age at arrival = 37 days, *n* = 15 rural; average age at arrival = 40 days, overall, 19 males, 11 females).

##### Year 3—2019

Twenty-one mother-pup pairs were captured at 2 rural colonies and 1 urban colony (*n*=11 urban; average age at arrival = 19 days, *n*=10 rural; average age at arrival = 12 days). To further exclude any effect of individual experience (even when carried by their mothers), in this season, we either caught pregnant mothers (who gave birth in the lab) or mothers with very young pups—the average forearm length was 48.7±10.0 mm accounting for an age of 14 days on average. Egyptian fruitbat pups are born with closed eyes and folded ears until they reach approximately 10 days of age [65], meaning that their sensory systems were at their most underdeveloped stage limiting experiences of their environment prior to arriving at the lab.

In both years 2 and 3, city and rural bats were housed separately in identical rooms (~ 2.5 × 2 × 2.5 m^3^) with a (12:12) fluctuating day/night light cycle and a regulated temperature of 27 °C. Across all years, bats were weighed and scaled weekly to keep track of their growth and health. The fur of all individuals was bleached with unique identification marks, and an experimenter checked twice a day that all mother/pup pairs were together. Bats were provided with fresh fruit ad-lib daily, including watermelon, banana, apple, and melon.

#### The foraging box-test

**Fig. 2:**
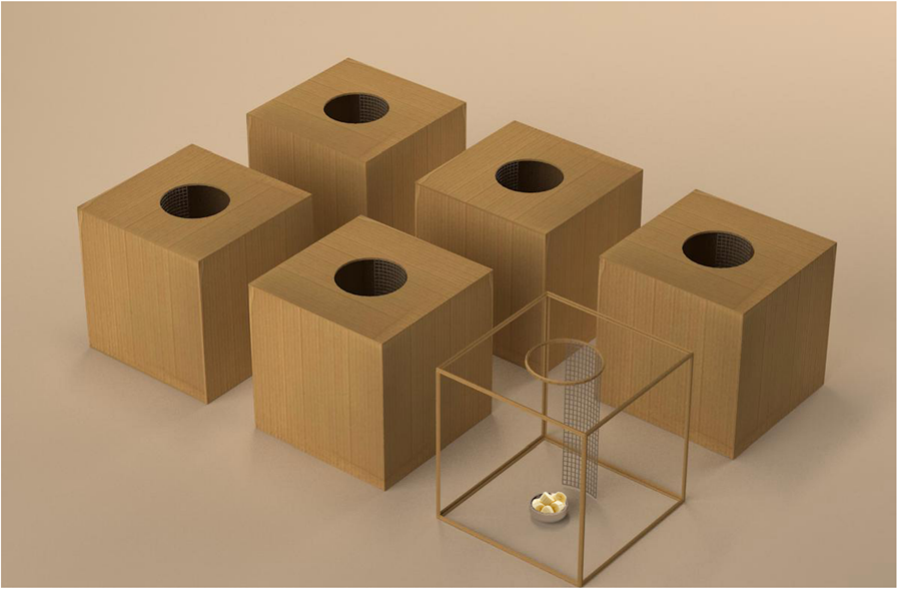
The foraging box-test setup. Each box had a hole with a ladder leading inside to a bowl with 25 pieces of fruit (150 g) + nectar (50 ml). During the basic setup sessions, all six boxes contained an accessible food bowl. The learning sessions were set up identically, but with only one box containing an accessible food source and the remaining boxes containing a food source covered with mesh (thus generating similar odor cues but inaccessible)

Behavioral assays were conducted in order to assess individual personality traits. The experimental apparatus consisted of a tent (3.9 × 2.7 × 1.9 m^3^) placed inside a room, with six, identical plastic boxes placed on its floor (i.e., foraging boxes, 64 × 38 × 40 cm^3^). Each box had an entry hole (10 cm diameter) with a mesh ladder leading to a food source consisting of daily fresh seasonal fruit (i.e., banana, apple, watermelon, and melon with mango juice, Fig. 2). Boxes containing fruit were washed between trials. We used two identical experimental tents (positioned in different rooms) to nightly test two juvenile bats (one rural and one urban) simultaneously. Bats were tested individually overnight (for 12 h between ~ 16:00 and 04:00) and they were removed from the room on the next morning. The experiments were recorded using an infrared video camera (Sony HDRCX730, Sony FDR-AX53), using an infrared light situated outside the tent to light the room homogeneously (Methaphase Technologies Inc-ISO-14-IR-24).

#### Experimental procedure

Experiments were carried out across the three years. At the beginning of each experimental batch, groups of five urban/rural pups were separately exposed to the foraging box-test for one night. In this exposure night, each box contained 750 g of accessible fruit, enough to meet the nightly nutritional requirements of all participating bats together. The aim of this exposure was to let the juvenile bats explore the foraging boxes and learn to feed from them, and thus to exclude effects of neophobia in the latter experiments.

##### The basic setup

Following the exposure session, each pup participated individually in between 1 and 3 sequential full-night sessions across consecutive nights (Additional file 1: Table S1. In the basic setup, each of the 6 boxes was accessible via a ladder, containing the same amount of excess food. Each box had 25 pieces of fruit + 50 ml nectar, which amounts to ~ 150 g and is enough food for a single bat per night to avoid any effects of food depletion. We used this paradigm to assess risk-taking and exploratory tendencies (see below).

##### The learning setup

This paradigm, used the same 6 box setup as above, only that now five out of the six boxes contained a non-accessible, food source, covered with mesh, providing similar olfactory cues but not allowing bats to reach the fruit. We tested the pups’ ability to learn the changing conditions—they had to reverse their previous learning that all boxes offer available food.

In all experiments, at the beginning of the session, the pup was placed for approximately 30 min in a familiar carrying bag (42 × 26 × 35 cm^3^) to acclimate. The bag with the bat was then placed at the circumference of the tent with its opening facing the foraging boxes. The pup could exit the bag voluntarily. The order of pups used in the experiments was determined by age, with older pups tested first, to minimize age differences between the pups during the experiments. Urban and rural pups of the closest age were tested at the same time as much as possible. Pups on average were 91.3±38.5 days old when participating in their first exposure experiment.

##### Cross-fostering experiments

To examine whether the differences in personality traits between urban and rural pups were acquired or innate, in the third year, we performed cross-fostering experiments.

We cross-fostered 11 mother-pup pairs (6 rural and 5 urban, Additional file 1: Table S1). Each cross-fostered pup was detached from its biological mother and transferred to the nipple of a mother originating from a different environment of origin (i.e., urban pups were transferred to rural mothers and vice versa). Following the switch, cross-fostered mother-pup pairs were left in a small carrying bag for approximately a week for bonding, checked hourly to assess that the pup was being fed and cared for by its adoptive mother. Following this bonding period, cross-fostered pairs were placed back in their respective (urban/rural) colony based on the origin of the adoptive mother. Cross-fostering was carried out at a very young age, when pups were non-volant and completely dependent on their mothers for survival (45±4 mm forearm on average, equivalent to an age of 10 days at most). These pups were tested in their first foraging box-experiments on day 106±16 on average, thus allowing ample time for maternal effects to take place.

#### Behavioral analysis

Video recordings of each session were independently watched and scored by 2 observers, to account for human error. A max of 5% of scoring events were in disagreements between the observers and were resolved by a third observer, who re-examined the disagreement and ruled. Scoring included annotating landings and entering of the boxes including the time and duration of each event, and the box number. This scoring was used to assess the following personality traits.

##### Risk-taking/boldness

the box entry-to-landing ratio, that is the proportion of landing events that ended in box entries was used as a proxy of boldness. This measure was previously found to be strongly associated with a vigilant head-up posture accompanied by substantial ear movements that are typical for scanning the environment for potential threats or dangers in this species.

##### Exploration

The proportion of boxes visited at least once was used as an estimate of exploration. Note that each box contained enough food for the entire night, so there was no need to test more bats.

##### Learning

To assess how individuals learn to adapt to the changes, we assessed the average number of times a bat re-sampled a foraging box after experiencing that food was not accessible there. In this experiment only one box was accessible, the location of the open box was kept constant across all experiments.

#### Milk cortisol

Milk samples were collected from 31 wild caught lactating bats originating from urban and rural colonies (*n*=17, urban colony: Herzliya, *n*=14, rural colony: Beit Guvrin; respectively, Additional file 1: Table S1). Samples were collected 2 h post-capture, providing sufficient time for negative feedback to both kick in and ramp down cortisol secretion [66–68]. Furthermore, full mammary evacuation occurred within 2–5 min, meaning another acute release of cortisol may have not yet kicked in [67, 69]. We thus argue that the cortisol levels evaluated, represent the return to a post-recovery (close to baseline,). Collection was carried out separately from each mammary by gentle hand stripping of the nipple. To minimize sampling bias, each mammary was fully evacuated as indicated by the transition from streaming milk to solitary droplets of milk during hand collection. Full mammary evacuation occurred within 2–5 min for all subjects.

For consistency, all samples were collected, by a single researcher 2 h post capture. The samples were stored frozen at − 20 °C until milk composition analysis. Following milk collection mother-infant pairs were returned to their respective colonies.

To extract cortisol from bat milk, we used solid-phase extraction (SPE) with C18 columns, which has been shown to yield a high and consistent steroid recovery by removing potentially interfering lipids in a variety of tissue types [70, 71]. Briefly, 50 μl of milk was incubated in 500 μl of ice-cold 80% HPLC-grade methanol (MeOH) overnight at 4 °C. Samples were then centrifuged (3000*g* for 10 min) and supernatant was collected and added to 5 mL of deionized water, prior to loading on carbon-bonded silica C18 filter column cartridges (Agilent Technologies, Santa Clara, USA) on a vacuum manifold. Columns were first primed with 5 ml of 100% ethanol, equilibrated with 10 ml deionized water before loading the diluted 5 mL sample. Next, 10 ml of 40% MeOH was used to remove lipids (e.g., triglycerides, cholesterols, and fatty acids) that could interfere with the cortisol assay [72]. Cortisol was then eluted using 5 ml of 90% MeOH and these samples were dried in a speed vacuum concentrator (Thermo Fisher Scientific Inc, Pittsburgh, USA) at 60 °C for 4 h. All SPE extractions included a solvent blank as a negative control. Dried extracts were stored at − 20 °C until assayed for cortisol.

Cortisol concentration was quantified using an enzyme-linked immunosorbent assay (ELISA) kit (Arbor Assays Inc, Ann Arbor, USA). Pooled milk samples were used to validate parallelism of a serial dilution with the assay standard curve, test the recovery of exogenous cortisol, and assess the removal of endogenous cortisol using dextran-coated charcoal (Additional file 1: Figure S3). All samples and standards were run in duplicate. Assay sensitivity was 24.1 pg/ml, and the intraassay coefficient of variation was 7.4% (*n* = 31 samples). Note that cortisol measurements likely represent maternal circulating levels, as mammary glands are not thought to synthesis it directly [73].

### Statistical analysis

Mixed effect GLMs were used to compare urban and rural behavioral traits from all trials using MATLAB (R2018a, MathWorks Inc.). For every behavioral parameter examined (i.e., risk-taking, exploration, and learning), the origin (urban vs. rural), fostering condition (biological vs. adoptive mother), and their interaction was set as fixed effects while the bat’s ID and the year of the experiment were set as random effects. When examining the effect of pup age and sex on behavior, these variables were added as another fixed effect (*n*=86). We used the logit link function because we tested proportions.

To examine whether cross-fostered pups resembled their biological or adoptive mothers, we carried out Pearson’s correlation tests between the average risk-taking and exploration indices across repetitions of cross-fostered pups with their biological and adoptive mothers (*n*=11, *n*=7; respectively). Initially, there were 11 biological mothers of cross-fostered pups; however, 2 pups died in their first week of life and were replaced with orphaned pups from the same origin (so the number of pups did not change, but we could not test their biological mother). An additional mother escaped before testing and the remaining mother was removed from the experiment due to health issues.

To assess individual consistency in both risk-taking and exploration, we carried out Pearson’s correlation tests between all three repeats of the basic set-up for both mothers and pups (pups: trial1 *n*=48, trial 2 *n*=48, trial 3 *n*=18; mothers: trials 1, 2, and 3, *n*=21; Additional file 1: Table S1).

## Supplementary Information

**Additional file 1: Figure S1.** Adult urban bats are more prone to risk-taking than rural bats. **Figure S2.** Pups are temporally consistent in their risk-taking and exploratory tendencies. **Figure S3.** Assay validations for the measurement of cortisol in Egyptian fruit bat milk. **Table S1.** Egyptian Fruit bat pups that were captured together with their mothers in natural rural and urban colonies between September-October 2017, April-May 2018, and April-May 2019. Each bat was brought to Zoological Garden in Tel Aviv University with their respective mothers.

## Data Availability

The datasets supporting the conclusions of this article are available in the drobox repository: https://www.dropbox.com/sh/n8kyf3illrk7zzs/AAAIoqOJgEikXPVeCAta1Wiua?dl=0 [74]
