# Supplementary material for: Urban bat pups take after their mothers and are bolder and faster learners than rural pups
Source: BMC Biol. 2021 Sep 7;19:190. doi: 10.1186/s12915-021-01131-z (PMC8422611; doi:10.1186/s12915-021-01131-z)
Supplement: Supplementary file 1 — Additional file 1: Figure S1. Adult urban bats are more prone to risk-taking than rural bats. Figure S2. Pups are temporally consistent in their risk-taking and exploratory tendencies. Figure S3. Assay validations for the measurement of cortisol in Egyptian fruit bat milk. Table S1. Egyptian Fruit bat pups that were captured together with their mothers in natural rural and urban colonies between September-October 2017, April-May 2018, and April-May 2019. Each bat was brought to Zoological Garden in Tel Aviv University with their respective mothers. [file 12915_2021_1131_MOESM1_ESM.docx]

**Additional File 1 - Supplementary Materials for:**

**Urban bat pups take after their mothers and are bolder and faster learners than rural pups**

**Authors:** Lee Harten^,1^, Nesim Gonceer ^,1^, Michal Handel^1^, Orit Dash^1^, H. Bobby Fokidis^2^ & Yossi Yovel^1,3^

^1^Department of Zoology, Faculty of Life Sciences, Tel Aviv University, Tel Aviv 69978, Israel.

^2^ School of Life Sciences, Arizona State University, P.O. Box 874601, Tempe, AZ 85281, USA.
^3^Sagol School of Neuroscience, Tel Aviv University, Tel Aviv 69978, Israel.

Correspondence to Yossi Yovel:

yossiyovel@gmail.com

**This file includes:**

Figs. S1 to S3

Tables S1


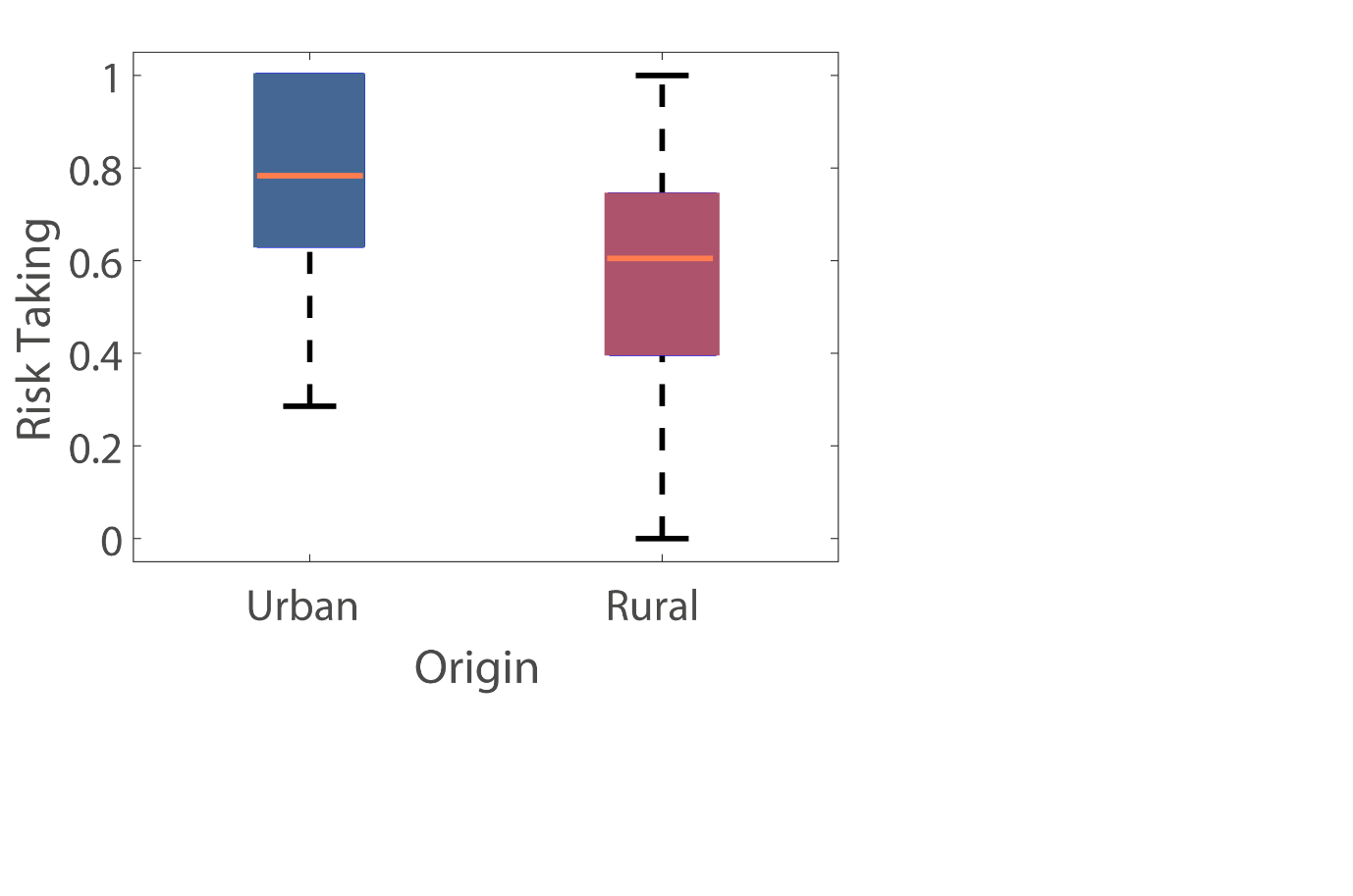


Figure. S1. Adult urban bats are more prone to risk-taking than rural bats. Boxplots show: Risk-taking (box entrance to landing ratio) of adult males as a function of their origin: urban (blue) vs. rural (red). Box plot lower and upper box boundaries show the 25th and 75th percentiles, respectively, with the median inside. The lower and upper error lines depict the 10th and 90th percentiles, respectively. Urban bats were significantly more prone to take risks than rural bats on their arrival in the lab, (n= 20 urban vs. 20 rural bats', 0.74±0.24 vs. 0.58±0.25, P=0.004; GLM with risk-taking set as the explained variable, the population origin (rural or urban) as a fixed effect and the specific colony and time-batch set as random effects). 40 adult male Egyptian fruit bats (*Rousettus aegyptiacus*) were caught in their roosts in three batches between Jul 2016 - Jan 2017. Urban bats (N=20) were brought from 3 different roosts that are located inside cities (Herzliya and Ramat Gan) while rural bats (N=20) were brought from 3 rural roosts (near Beit Govrin, Shoham and Sgafim). These 40 bats (20 urban and 20 rural) were run through behavioral experiments after which they were released back to their colony of origin. Wild bats cannot be easily aged, but we always tried to choose healthy adults. Bats were marked for individual recognition. The urban and rural bats in each batch were housed in two separate but identical rooms, housing conditions were identical to those described in housing description for the main study. Adult males participated in a one hour basic-setup trial each, in a third room (3.9m*2.6m*2.3m, same room for all bats), with the basic box setup identical to those for the main study. During this time the bats landed at least 10 times each (with an average 18.4±4.5 times), which allowed us to assess their behavior. These experiments were video-monitored and the same risk-taking behavioral parameters as for the main study were extracted. Generalized linear mixed effect models were used to compare urban and rural risk-taking behavior using MATLAB (R2015a, MathWorks inc.), the population (urban vs. rural) was set as the fixed effect while the specific colony were set as random effects. Preliminary experiments were approved by the Tel-Aviv University IACUC – permit number 04-16-063

**Figure S2. Pups were consistent in their** (A) Risk-taking (B) Exploratory tendencies across trials. A-B Correlation matrices where each cell denotes the Pearson correlation coefficient between two basic setup sessions (denoted B1, n=48; B2, n=48; B3, n=18). The color indicates the strength of the correlation. All correlations were significant for both risk-taking and exploration (P < 0.02, (P < 0.004); respectively).

**
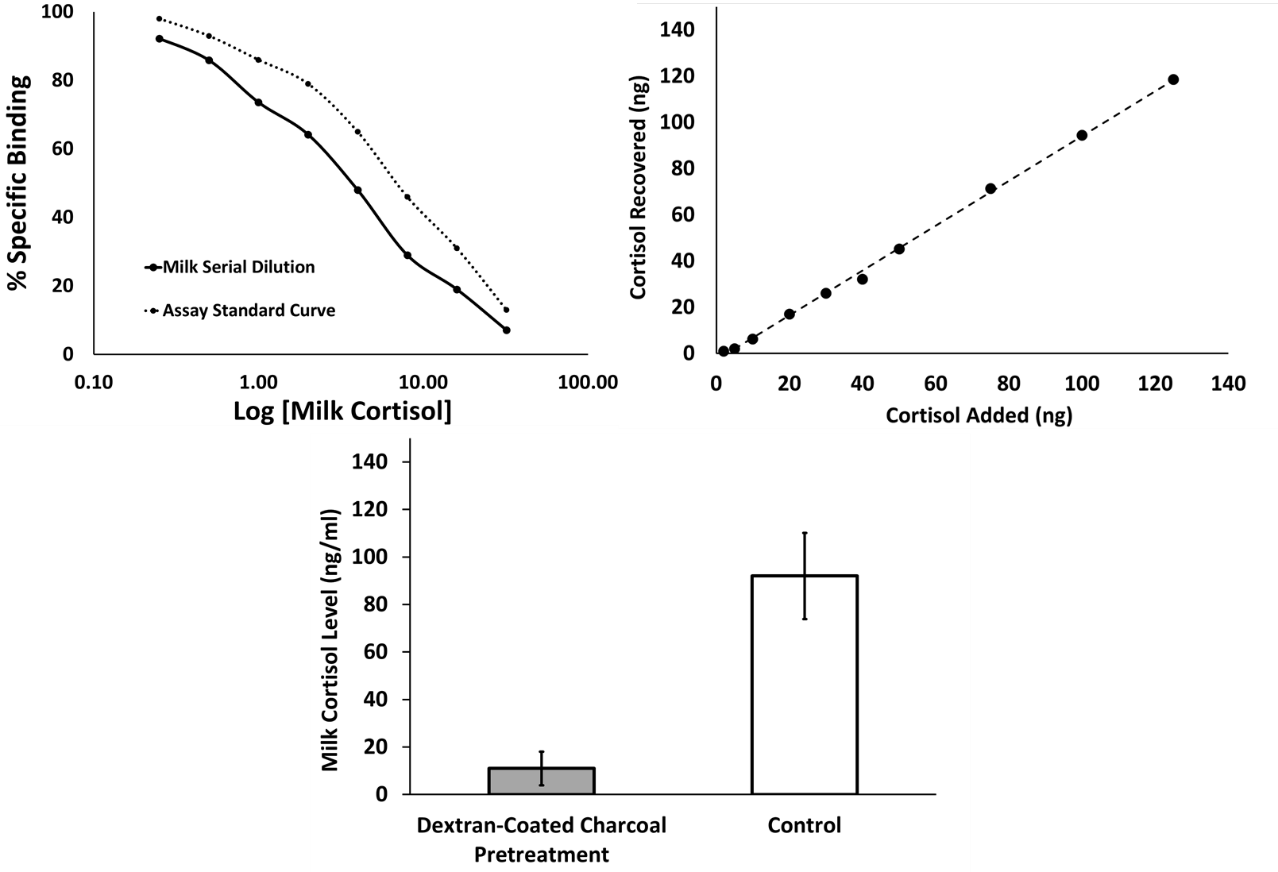
**

**Figure S3. Assay validations for the measurement of cortisol in Egyptian fruit bat milk.**  (A) Serial dilution of a pooled milk sample was model using linear regression (R^2^ = 0.903; F_1, 7_ = 35.634, P < 0.001) and had the same slope (i.e., was parallel) to the cortisol assay standard curve (P = 0.476) (B) Significant correlation between milk samples spiked with varying concentrations of exogenous cortisol and their subsequent recovery (r = 0.961, P < 0.001); and (C) pooled milk samples incubated overnight in the presence of dextran-coated charcoal exhibited reduced cortisol compared to untreated controls.

***Urban and Rural natural colonies in Israel***

***Table S1:*** Egyptian Fruit bat pups that were captured together with their mothers in natural rural and urban colonies between September-October 2017, April-May 2018, and April-May 2019. Each bat was brought to Zoological Garden in Tel Aviv University with their respective mothers.

| Season | Rural/Urban | Colony ID | N pairs | Coordinates | Experiments (repetitions) |
| --- | --- | --- | --- | --- | --- |
| 2017 | Rural | Herzliya | 35 | 32°10'18.4"N  34°48'51.1"E | Basic setup (1)  Learning (1) |
| 2018 | Rural | Beit Guvrin | 4 | 31°36'45.8"N  34°53'41.7"E | Basic setup (2)  Learning (1) |
|  |  | Sgafim | 11 | 31°68'37"N  34°91'089"E |  |
| 2018 | Urban | Herzliya | 7 | 32°10'18.4"N  34°48'51.1"E |  |
|  |  | Shoncino | 3 | 32°03'59.2"N  34°47'14.1"E |  |
|  |  | Yafo | 1 | 32°03'18.1"N  34°45'14.4"E |  |
|  |  | Hayarkon Bridge | 4 | 32°05'45.0"N  34°48'11.6"E |  |
| 2019 | Urban | Herzliya | 10 | 32°10'18.4"N  34°48'51.1"E | Basic setup (3)  +  Cross-fostering |
| 2019 | Rural | Beit Guvrin | 8 | 31°36'45.8"N  34°53'41.7"E |  |
|  |  | Tinshemet | 3 | 31°59'43.2"N  34°57'19.2"E |  |
